# Involving service users to identify research priorities in a UK forensic mental health service

**DOI:** 10.1192/bjb.2020.131

**Published:** 2021-12

**Authors:** Anne Aboaja, Bunny Forsyth, Helen Bates, Robert Wood

**Affiliations:** 1 Roseberry Park Hospital, Tees, Esk and Wear Valleys NHS Foundation Trust, UK

**Keywords:** Research priorities, patient and public involvement, forensic mental health services, service users, involvement

## Abstract

**Aims and method:**

Patient and public involvement (PPI) is a priority for health research. PPI improves the relevance and quality of research. The study aimed to involve service users in identifying research priorities for the service. A two-phase adapted Delphi technique was used to generate a list of research topics from service users in secure in-patient mental health settings and on specialist mental health prison wings. Topic content analysis was undertaken. Service users were further consulted, and research themes were ranked in order of priority.

**Results:**

Of the eight research themes identified, the three given the highest priority by service users were, in descending order, physical health, future plans and moving on, and causes of illness and crime.

**Clinical implications:**

Service users are willing to be involved in setting research priorities for mental health services. Through non-tokenistic PPI, service users can uniquely shape the research agenda of mental health services.

Patient and public involvement (PPI) is a strategic priority for the National Institute of Health Research,^1^ enabling stakeholders to have roles other than as research participants throughout the research process.^2^ Despite the barriers,^3^ challenges of time and methodological conflict, PPI can bring benefits to research.^4^ It improves the relevance and quality of research,^1^ and increases recruitment success.^5^ Researchers in this field have evidenced the value that the PPI perspective can bring to research,^6^ but caution using tokenistic approaches. Utilising a devolved model of working with user-led organisations or groups can support effective involvement,^7^ as long as all parties clearly understand the underpinning philosophy. A related approach of using a reference group specifically established to guide and assist with the conduct of a research project is also recognised as valuable.^8^

The Tees Esk & Wear Valleys NHS Foundation Trust's forensic directorate aims to significantly increase research activity. Although forensic mental health priorities have been previously proposed, they have arisen from studies that did not include service users,^9,10^ focused on specialist areas outside of in-patient^11^ or prison settings,^12^ and sought consensus between service users and professionals.^11^ This paper describes how non-tokenistic PPI through adapted Delphi methodology can be applied in a forensic service, to identify service user priorities for research.

## Method

### Setting

The forensic service has 18 in-patient clinical areas, offering 224 medium-secure, low-secure and locked rehabilitation beds to adult males and females with a range of mental disorders, intellectual disabilities (also known as learning disabilities in UK health services) and autism spectrum disorders. The service also provides mental healthcare to 11 prisons in the north of England and to patients in the community. Within the prison system, there are 29 beds across two specialist mental health wings (clinical areas). Each of the 20 clinical areas holds a regular service user-led community meeting with staff support. The meeting is used to share information, elicit views that may be passed on to service managers and discuss items of interest to the in-patient experience. Attendance and participation at these meetings is voluntary. There is also a community service that does not have equivalent service user community meetings.

### Ethical approval and service user involvement

No identifiable participant data were collated, and all data were collected through existing service user meetings. Ethical approval was not required for this study, which was accountable throughout to the Forensic Speciality Development Group in Tees, Esk and Wear Valleys NHS Foundation Trust. The project proposal was approved by the hospital-wide service user group, whose suggestions shaped study design.

### Design

The Delphi technique is an established methodology that has been used widely in psychiatry to consult with and build consensus among experts in the field.^13^ A two-phase modified Delphi exercise was undertaken in which service users were consulted as experts by experience of the forensic service. An iterative process was followed to obtain consensus on the most important research themes.

#### Participants

All users of services who were available and willing to attend an established community meeting in one of the clinical areas were eligible to participate in the exercise. Service users in prison who were not on a specialist mental health wing and users of forensic community mental health services were therefore not eligible to participate.

#### Delphi process: phase 1 – obtaining the initial views of service users

A consultation form was developed for use in community meetings. The form comprised three sections. In the first section, a succinct definition of research and examples of health research questions unrelated to mental health were given; for example, ‘what causes heart attacks?’, ‘what is the cure for cancer?’ and ‘which shampoo will make your hair grow faster?’ The second section provided information about the purpose of the exercise, rationale for consulting service users, the constitution of the project team, anonymisation of responses given and the extent to which the findings would be shared. In the final section, three prompt questions were listed to elicit relevant responses about research priorities:

- What questions should researchers in this service try to answer?
- What discovery would you most like researchers to make?
- What do you think we need to know more about in forensic services?

The consultation form was emailed to a senior nurse in each clinical area, with a cover letter explaining the background to the project and how to use the form to elicit and anonymously record service user views. Instructions were given to record the numbers of patients on the ward, at the meeting and contributing to the process. The importance of service user voluntariness in participating in the exercise was highlighted. The senior nurse was asked to present the form during a community meeting and invite service users to answer the three questions. Answers were written down on the form, which was returned the project administrator.

#### Delphi process: phase 1 – analysing the initial views of service users

Two consultant psychiatrists and an in-patient nurse from the project team jointly undertook content analysis^14^ of all responses, by hand. Disagreements were resolved through consensus. An inductive–deductive approach was used to move from a large number of specific research questions and objectives provided by participants to a smaller number of general research topics.^14^ Each individual response was read and grouped into one category with similar responses. Each category was given a title that summarised the content of the associated responses. Then, responses in each category were reviewed in turn, to consider whether it would be more appropriate to place each response in an alternative category. This step ensured that each category accurately reflected the content of the respective responses. Next, categories that were considered similar were combined. Category titles were broadened to incorporate very small groups and single responses that could not be grouped elsewhere. Principles for the iterative analysis were to avoid single-response groups, to accurately represent service users’ responses and to identify between approximately five and ten themes. This target number of themes was chosen to ensure the service user voice was not lost through excessive combining of responses, resulting in a small number of themes. Similarly, the aim was to avoid a very long list of themes, which would be less useful in identifying the specific areas of research that should be prioritised. The final categories represented an unranked list of research priorities.

#### Delphi process: phase 2 – obtaining prioritised views of service users

The results of the analysis were listed as themes on a follow-up consultation form, which defined research, explained the purpose of the project, described the first phase of the Delphi process and highlighted the voluntariness of participation. To avoid responder bias associated with the ordering of the themes, the themes were displayed in a circle on the follow-up consultation form. The form and a cover letter were sent to a qualified member of staff in each clinical area who had responsibility for disseminating research information (the Research Champion). The cover letter provided instructions on how to obtain anonymised responses from service users during the community meeting. Research Champions offered copies of the form to willing service users present at the community meeting, who were invited to circle their top three priority areas for research. The Research Champion returned completed forms to the project administrator.

#### Delphi process: phase 2 – analysing the prioritised views of service users

Two consultant psychiatrists, a ward-based staff nurse and a senior nurse working in the prison service met to analyse the completed follow-up consultation forms from phase 2. The themes identified in phase 1 were listed on a whiteboard. Each response form was considered in turn by the analysts, jointly. A mark was added alongside each theme on the whiteboard every time that theme was circled as a priority theme on a response form. After reviewing all responses, frequency counts were calculated for each theme. The themes were then ranked according to the number of service users who had considered each theme to be one of their top three research priorities. The overall top three research priorities were highlighted.

## Results

### Participation in the Delphi process

Service users from 20 clinical areas (18 wards and two prison wings) were eligible to participate in each phase of the Delphi process. Some clinical areas did not provide details of the number of service users who attended the community meeting or proposed priority research areas in phase 1. Therefore, the overall response rate in phase 1 was calculated with the number of participating clinical areas rather than individual service users.

Of the 20 clinical areas invited to participate in phase 1, responses were received from six clinical areas (five wards and one prison wing) (Table 1). In phase 2, five clinical areas, including one prison wing, returned completed follow-up consultation forms from 27 service users (Table 1). Based on the number of beds in the service (*n* = 253), this is equivalent to 10.7% of the total number of eligible service users.

**Table 1:** Participation of service user clinical areas in phases of the Delphi process

	Participation in phase 1	Participation in phase 2	Participation in both phases	Participation in at least one phase
Number of clinical areas responding	6 (30%)	5 (25%)	1 (5%)	10 (50%)
Number of clinical areas not responding	14 (70%	15 (75%)	19 (95%)	10 (50%)
Number of clinical areas invited to participate	20 (100%)	20 (100%)	20 (100%)	20 (100%)

There was overlap of clinical areas participating in the two phases of the Delphi process, with service users from one (5%) of the 20 clinical areas participating in both consultation phases. Although most services users were not involved in both phases, service users from half (*n* = 10, 50%) of all clinical areas, including both prison wings, participated in at least one of phases of the Delphi process (Table 1).

### Profile of service users participating in the Delphi process

Male and female service users from both prison and in-patients clinical areas participated in the study (Table 2). Input during at least one of the Delphi process phases was received from service users within hospital-based clinical areas of all three levels of security provided by the forensic service. However, females in the locked rehabilitation clinical area were not involved in the study. Services users with a mental disorder, as well as those with an intellectual disability or autism, participated in the Delphi process.

**Table 2:** Description of the clinical areas in which participating service users resided

Clinical areas involved in the Delphi process of identifying research priorities		Male	Female
Prison		Yes	Yes
In-patient	Medium security	Yes	Yes
	Low security	Yes	Yes
	Locked rehabilitation	Yes	No
	Mental disorders	Yes	Yes
	Intellectual disabilities and autism	Yes	Yes

### Identification of research themes that are important to service users

Service users offered 63 suggestions for research in the first phase of the Delphi process (Table 3). The suggestions were written in a combination of questions and statements. Eight research themes were identified through thematic analysis (Table 3). The second phase of the Delphi process revealed how 10.7% of all service users across the forensic service prioritise these themes. The top three priorities for research are, in descending order, physical health, future plans and moving on, and causes of crime and illness. Other themes of importance are treatment and cures, length of stay, trust and attitudes, purpose of life and dealing with change.

**Table 3:** Research priorities as ranked by service users

Examples of responses received in Phase 1	Themes identified in phase 2	Ranking in descending order of priority
Best way to lose weight?	Physical health	1
The correlation between medication and physical health well-being		
My metabolism has slowed, how do I get it going again?		
Best way to lose weight off the stomach?		
How does being in a forensic service affect your chances of getting a job?	Future plans and moving on	2
Anxiety about leaving [prison mental health service]		
Will I always need medication?		
What causes offenders to reoffend?	Causes of crime and illness	3
We need to know more about autism		
Information on eating disorders		
Is there a cure [for] self-harm?	Treatment and cures	4
We should make treatment shorter (DBT [dialectical behaviour therapy] is too long)		
Could we cure LD [intellectual disability]?		
To have a tablet that cures everything		
We should try to make people's stay in hospital shorter, not waiting for treatment	Length of stay	5
Discover the length of time you are going to be in hospital		
Attitudes of staff in prison	Trust and attitudes	6
Trust issues with officers		
Attitudes of healthcare staff…they make me nervous		
More about what you want from life	Purpose of life	7
What is the purpose of life?		
Why are you born to die?		
Changes in staff is destabilising	Dealing with change	8
How to cope with change		
The impact of staff leaving [prison mental health service]…it's difficult to have staff change so frequently		

## Discussion

Research that is to have a meaningful impact on the care, experience and recovery of those who use forensic mental health services must involve service users from the start of the research cycle, at the point of setting research priorities. First, this study showed that PPI research methodology was effective in involving some, but not all, male and female service users in both prison and hospital settings who have a mental disorder, intellectual disability or autism. Second, through this adapted Delphi approach, services users identified eight research priorities for forensic mental health and intellectual disabilities.

It is notable that service users place a high value on health research with a holistic conceptualisation of health, including physical, mental and spiritual (existential/‘purpose of life’) domains. The list indicates that service users with a history of mental disorder and offending are interested not only in obvious aspects of forensic mental health, such as mental illness, crime and treatment, but also in staff relationships (‘trust and attitudes’) and the aetiology of their difficulties.

### Contextualisation of findings

In the present study, physical health was ranked as the top priority, with a focus on weight loss. Surprisingly, this theme did not feature in the lists generated from earlier exercises to establish the research priorities in forensic mental health.^9,11,12^ The explanation for this notable difference may lie in the recent incentivised drive by commissioners of secure mental healthcare in England for providers to take demonstrable steps to improve physical health, particularly through achieving a healthy weight.^15,16^

There was overlap with the findings of a previous study showing that service users, as well as professionals, prioritise epidemiological research into factors associated with crime and recidivism, and research oriented toward recovery topics such as the future use of mental health services and employment.^11^ Further consistency was found in the high priority given by both clinicians and service users to research about effective treatments and interventions.^10–12^

This finding of common interest is not surprising, given the partnership nature of many treatments involving the professional, who delivers, prescribes or administers the treatment, and the service user, who accepts or refuses the treatment that may cause harm, benefit or no effect. However, although previous studies mentioned treatment as a research priority, service users in the present study clearly linked treatment to cure. Current understanding among mental health clinicians and academics about the nature of disorder, disease, disability and concepts of recovery, diversity, social inclusion, person-centred care and stigma may partly explain why research questions such as ‘could we cure LD [intellectual disability]?’, which are important to some service users, are less likely to be posed by professionals in a research priority-setting exercise.^17^

The advancement of risk assessment in forensic mental health is consistently reported as a research priority in studies based on literature review, professionals-only groups or mixed professional and service user groups.^9–11^ Although it is not clear why risk assessment did not feature as an important research area in the present study, which involved only service users, it is proposed that this topic might be of lesser importance to service users and greater interest to professionals, whose roles involve assessing and managing risk.

It is also notable that in contrast to a larger international study of research priorities for mental health and justice, the present study of service users lawfully detained in either a prison or secure hospital under the Mental Health Act 1983 did not recommend research into legislation and policy.^11^ Length of stay in hospital emerged as one of the new research priority areas in the present study. Although it may appear surprising that this was not a theme, given priority in previous studies, it may be that addressing other themes widely reported, such as treatment, may ultimately have an effect on overall length of stay.^18^

### Strengths and limitations

It might appear that the responses of service users point to areas that have been extensively researched. For example, much is already known about effective weight loss interventions in the general population. Although such responses may reflect limitations of the phrasing of questions used to elicit the initial views of service users, they may also reveal a lack of evidence of context-specific effectiveness and acceptability of interventions in forensic services.^15^ This is evident by the aforementioned current emphasis on finding effective ways to achieve weight loss among service users in secure mental health settings.

The use of existing consultation structures is recommended for ascertaining the research priorities of service users within forensic mental health services.^19^ The community meeting was, therefore, an appropriate forum in which to obtain the research opinions, because service users were already accustomed to voluntarily making suggestions to improve the service in this group setting. In contrast, a formal panel meeting may have been less accessible to some in-patients and prisoners.^19^ Although the presence of staff and other service users might have influenced responses provided, the wide range of individual answers given, including criticism of staff attitudes, suggests that group bias was not significant.^20^

There is little evidence to guide sampling approaches in PPI, although convenience sampling is most commonly used.^21^ The modest response during both phases of the study is a significant limitation, and may reveal lack of interest in or apathy toward research among the service user group. It is possible that such explanations may relate to psychopathology experienced by potential participants; for example, anhedonia in a depressive episode, or apathy as a negative symptom of schizophrenia. Alternatively, some service users may have limited understanding of research and the value of service user involvement in research. Unknown factors relating to the level of research interest among staff responsible for presenting the study to service users at community meetings may have contributed to the low response rate. Although the final list of priorities generated from the views of a small proportion of service users is valuable, it may not reflect the views of those service users who did not respond.

A strength of the study is effectively reaching a wide range of service users, with differing risk and health needs. The methodology of involvement was successful in increasing research involvement access to service users who, (because of reasons relating to health or risk) may not have had the opportunity to leave the ward or wing to attend a formal group meeting of service user volunteers, without limiting involvement to a select sample of existing service user representatives.^21^

Consideration was given from the outset to the evidence-based approaches to avoid tokenism and collaborate with user-led groups in a way that clearly explained the philosophy of the project. However, a formal reference research group of service users did not exist at the time of the study, although the generic (non-research) service user group was already established. Consultation with this service user group helped to shape the study design; ongoing consultation with the group may have been valuable in finding ways to increase the response,^5^ and to reduce sample bias.^20^ Although the involvement of professionals from in-patient and prison settings, as well as nursing and medical disciplines, ensured a healthy range of perspectives during the thematic analysis, service user involvement at this stage for collaborative data analysis would have further strengthened the study.^22^

### Implications and future directions

To our knowledge, this is the first study to use PPI principles and the Delphi technique to establish research priorities from the exclusive perspective of service users within a forensic service for mental health, intellectual disabilities and autism. The methods showed how a mental health service can overcome barriers^3^ and involve its users to identify priority areas of research. By contributing to research priority-setting exercises, service users demonstrated that they wish to express their views on the greatest research needs for forensic mental health services. It also revealed a willingness to be involved at the earliest stage of the research process, and a desire to influence the work of researchers in the field. Comparisons with similar studies highlighted the importance of understanding the service user perspective separately from that of professionals.

The eight research priorities were adopted immediately by the forensic service, serving as a checklist against which all proposed research is considered before service-level approval. Additional weight is given to proposed research in an area falling within one of the top three themes. Rarely should research be undertaken within the service that does not link directly or indirectly to this list. Embedding the service user perspective to this degree avoids tokenistic involvement,^6^ and allows service users, as experts by experience, to directly shape research strategy and influence future research. Findings have been shared with service users, senior managers and staff working across the forensic service. Although the scope of this study was limited to the service user perspective, further study is required to explore the extent to which the research priorities of clinicians working in this forensic service are aligned with those proposed by service users, and to understand any differences.

There is a lack of evidence to guide the best method of achieving engagement.^21^ There is benefit in exploring the enablers and barriers to PPI in research that involves service users in a forensic service. Early conversations should commence with representatives from community service users and carers/friends/families of service users, to develop appropriate methodologies for obtaining the views on research priorities from these two groups. The development of a carer research reference group may be an effective approach to carer involvement with this process.^8^

Further consultation with service users is required to develop a strategy to support ongoing involvement, ensuring that future research questions, methods and outcomes are acceptable and relevant to service users. The exercise of setting research priorities from the service user perspective could be repeated at 5-year intervals, to ensure that the service continues to prioritise research that is relevant to those who would benefit from the service. Given the expected benefits of PPI, the challenge is to evaluate the impact of this early service user involvement in setting research priorities, on subsequent phases of the research process and future service user involvement in, and engagement with, research.^23,24^

## About the authors

**Anne Aboaja** is a consultant forensic psychiatrist in the Forensic Service at Roseberry Park Hospital, Tees, Esk and Wear Valleys NHS Foundation Trust, UK. **Bunny Forsyth** is a consultant intellectual disabilities psychiatrist in the Forensic Service at Roseberry Park Hospital, Tees, Esk and Wear Valleys NHS Foundation Trust, UK. **Helen Bates** is an advanced nurse practitioner in the Forensic Service at Roseberry Park Hospital, Tees, Esk and Wear Valleys NHS Foundation Trust, UK. **Robert Wood** is a registered mental health nurse in the Forensic Service at Roseberry Park Hospital, Tees, Esk and Wear Valleys NHS Foundation Trust, UK.

## Data Availability

The data that support the findings of this study are available from the corresponding author, A.A., upon reasonable request.

## Supplementary material

For supplementary material accompanying this paper visit http://doi.org/10.1192/bjb.2020.131. click here to view supplementary material
